# 'Asking the Right Question'. A Comparison of Two Approaches to Gathering Data on 'Herbals' Use in Survey Based Studies

**DOI:** 10.1371/journal.pone.0150140

**Published:** 2016-02-25

**Authors:** James S. McLay, Abdul R. Pallivalappila, Ashalatha Shetty, Binita Pande, Moza Al Hail, Derek Stewart

**Affiliations:** 1 The Division of Applied Health Sciences, The University of Aberdeen, Aberdeen, United Kingdom, AB25 2ZD; 2 Pharmacy Department, Women's Hospital-HMC, Doha, Qatar; 3 The Department of Obstetrics and Gynaecology, The University of Aberdeen, Aberdeen, United Kingdom, AB25 2ZD; 4 The Department of Obstetrics and Gynaecology, Ninewells Hospital and Medical School, NHS Tayside, Dundee, United Kingdom, DD1 9SY; 5 Hamad Medical Corporation, Post Box – 3050, Doha, Qatar; 6 School of Pharmacy and Life Sciences, Robert Gordon University, Aberdeen, United Kingdom, AB10 7GJ; College of Tropical Agriculture and Human Resources, University of Hawaii, UNITED STATES

## Abstract

**Background:**

Over the last decade academic interest in the prevalence and nature of herbal medicines use by pregnant women has increased significantly. Such data are usually collected by means of an administered questionnaire survey, however a key methodological limitation using this approach is the need to clearly define the scope of ‘herbals’ to be investigated. The majority of published studies in this area neither define ‘herbals’ nor provide a detailed checklist naming specific ‘herbals’ and CAM modalities, which limits inter-study comparison, generalisability and the potential for meta-analyses. The aim of this study was to compare the self-reported use of herbs, herbal medicines and herbal products using two different approaches implemented in succession.

**Methods:**

Cross-sectional questionnaire surveys of women attending for their mid-trimester scan or attending the postnatal unit following live birth at the Royal Aberdeen Maternity Hospital, North-East Scotland. The questionnaire utilised two approaches to collect data on ‘herbals’ use, a single closed yes/no answer to the question “have you used herbs, herbal medicines and herbal products in the last three months”; and a request to tick which of a list of 40 ‘herbals’ they had used in the same time period.

**Results:**

A total of 889 responses were obtained of which 4.3% (38) answered ‘yes’ to herbal use via the closed question. However, using the checklist 39% (350) of respondents reported the use of one or more specific ‘herbals’ (p<0.0001). The 312 respondents who reported ‘no’ to ‘herbals’ use via the closed question but “yes” via the checklist consumed a total of 20 different ‘herbals’ (median 1, interquartile range 1–2, range 1–6).

**Conclusions:**

This study demonstrates that the use of a single closed question asking about the use of ‘herbals’, as frequently reported in published studies, may not yield valid data resulting in a gross underestimation of actual use.

## Introduction

**Fig 1:**
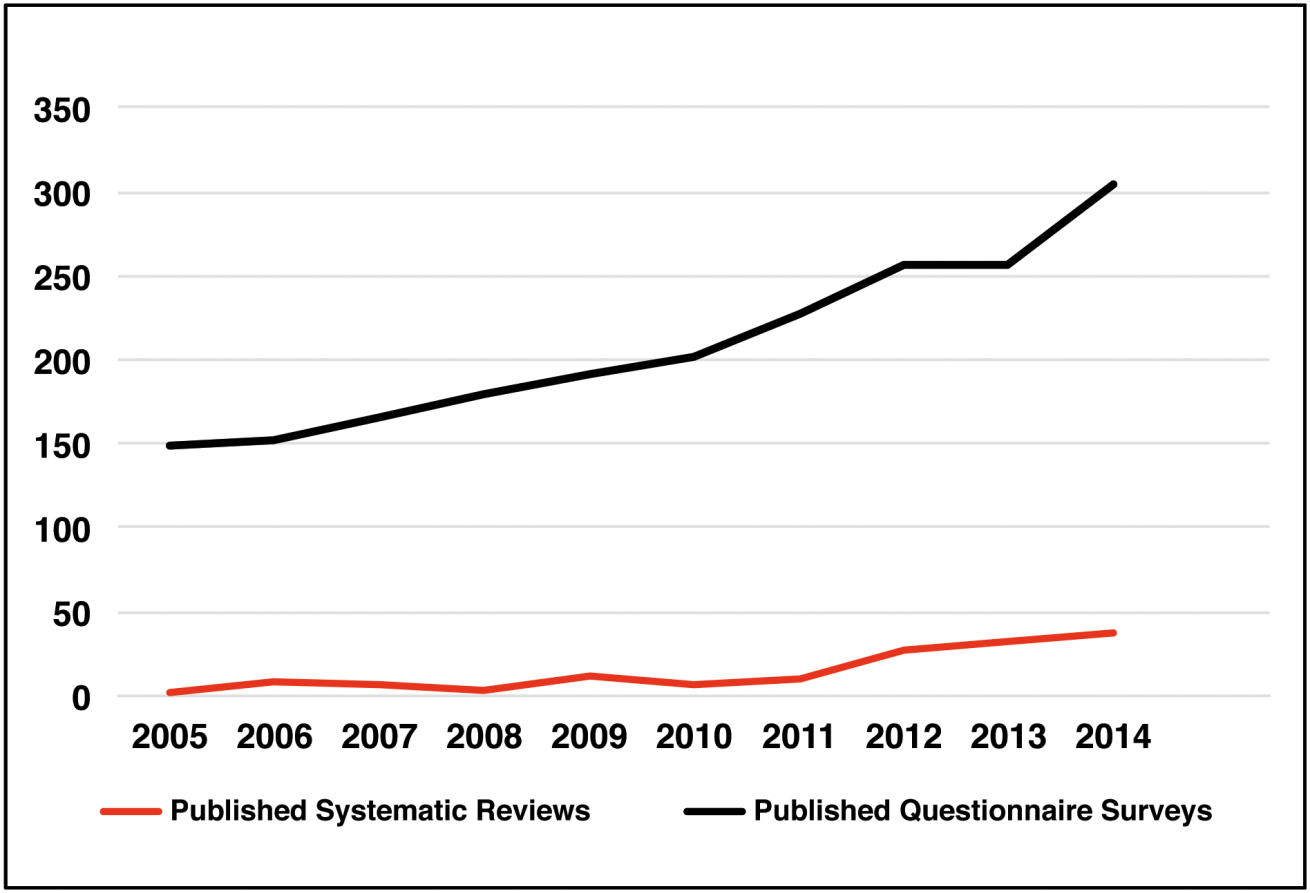
Number of hits over the last 10 years for Medline title search for ‘systematic review’ and ‘herb*’ and number of hits over the last 10 years for Medline abstract search of (‘questionnaire*’ or ‘survey*’) and ‘herb*’.

The increasing use of herbs, herbal medicines and herbal products (‘herbals’) to maintain health and treat a variety of medical conditions has generated significant academic interest. Since 2005 there has been a 20 fold increase in the number of systematic reviews and a two fold increase in prevalence surveys relating to ‘herbals’ published in peer reviewed journals (See Fig 1).

Given the paucity of robust data regarding the safety, efficacy and potential for interaction with prescribed medication, the widespread use of ‘herbals’ by the public should be of interest to the medical profession and regulatory authorities [1–4]. Of particular concern may be use by pregnant women, who may endanger both their own health and that of their baby via potential herbs-drug interactions or direct toxicity from active herbal ingredients or toxic adulterants [5–16].

A key limitation of most published studies reporting the use of ‘herbals’ is the failure to clearly define the scope of ‘herbals’ investigated. A recent systematic review of prevalence studies reporting the use of ‘herbals’ and other complementary and alternative medicine (CAM) modalities by pregnant women, identified that appropriate definitions of ‘herbals’ and CAMs were only provided in twelve of twenty-two studies [17]. Furthermore, detailed checklists naming specific ‘herbals’ and CAM modalities were described in only ten studies, with little uniformity in content across studies [17]. These failings limit direct inter-study comparison, generalisability, data pooling and the potential for meta-analyses.

This situation is not helped by the complex and major differences in the terms and definitions of ‘herbals’ originating from different regulatory authorities [18–20] (Table 1). In addition there are differences between countries in the regulatory classification of ‘herbals’. For instance in the United Kingdom some herbal products are classified as food supplements or cosmetics and others as medicines, while in the USA all herbal medicines and products are described as ‘Dietary Supplements’ [21–23].

**Table 1:** Definitions of herbs, herbal medicines, herbal products and Dietary Supplements used by the World Health Organisation, Medicines and Healthcare products Regulatory Agency (MHRA, United Kingdom) and the Food and Drug Administration (FDA, United States of America).

Term	Definition	Source
**Herbal Medicine**	Herbal medicines include herbs, herbal materials, herbal preparations and finished herbal products that contain as active ingredients parts of plants, or other plant materials, or combinations	World Health Organisation (WHO), 2000 (18)
**Herbs**	Herbs include crude plant material such as leaves, flowers, fruit, seed, stems, wood, bark, roots, rhizomes or other plant parts, which may be entire, fragmented or powdered	WHO, 2000 (18)
**Herbal materials**	Herbal materials include, in addition to herbs, fresh juices, gums, fixed oils, essential oils, resins and dry powders of herbs. In some countries, these materials may be processed by various local procedures, such as steaming, roasting, or stir-baking with honey, alcoholic beverages or other materials	WHO, 2000 (18)
**Herbal preparations**	The basis for finished herbal products and may include comminuted or powdered herbal materials, or extracts, tinctures and fatty oils of herbal materials. They are produced by extraction, fractionation, purification, concentration, or other physical or biological processes. They also include preparations made by steeping or heating herbal materials in alcoholic beverages and/or honey, or in other materials	WHO 2000 (18)
**Finished herbal products**	Herbal preparations made from one or more herbs. If more than one herb is used, the term mixture herbal product can also be used. Finished herbal products and mixture herbal products may contain excipients in addition to the active ingredients. However, finished products or mixture products to which chemically defined active substances have been added, including synthetic compounds and/or isolated constituents from herbal materials, are not considered to be herbal	WHO, 2000 (18)
**Herbal medicine**	A product is a herbal medicine if the active ingredients are herbal substances and or herbal preparations only	Medicines and Healthcare products Regulatory Agency (MHRA), Human Medicines Regulation, UK, 2012 (19)
**Herbal preparation**	A herbal preparation is when herbal substances are put through specific processes, which include: extraction, distillation, expression, fractionation, purification, concentration, fermentation.	MHRA, Human Medicines Regulation, UK, 2012 (19)
**Herbal substance**	The herbal substance being processed can be: reduced or powdered, a tincture, an extract, an essential oil, an expressed juice, a processed exudate (rich protein oozed out of its source).	MHRA, Human Medicines Regulation, UK, 2012 (19)
**Dietary supplement**	A product that: is intended to supplement the diet. Contains one or more dietary ingredients (including vitamins, minerals, herbs or other botanicals, amino acids, and certain other substances) or their constituents; is intended to be taken by mouth, in forms such as tablet, capsule, powder, softgel, gelcap, or liquid; and is labelled as being a dietary supplement.	Dietary Supplement Health and Education Act, USA (20)

As the majority of studies assessing the use of ‘herbals’ use a questionnaire based approach, it is essential that research participants clearly understand and correctly interpret the questions asked. Therefore terms used in the questionnaire should be explicitly defined to ensure the collection of valid data.

In an attempt to standardise data collection and reporting in CAM related studies, Quandt et al described the development of a CAM questionnaire (I-CAM-Q) to assess self-reported CAM use [24, 25]. The I-CAM-Q has four sections covering: visits to health care providers; complementary treatments received from physicians; use of herbal medicine and dietary supplements; and self-help practices. However, there is little specific reference to ‘herbals’ other than: ‘have you visited a herbalist in the last 12 months?’; ‘have you received herbs from a physician in the last 12 months?’ and ‘list up to three herbs/herbal medicines you have used in the last 12 months’. Of note there is inconsistency in the use of the terms ‘herbs’ and ‘herbal medicines’, there is no definition of these terms and there is no list provided of specific ‘herbals’ from which respondents may select. These are major issues which may impact the internal validity of the data.

Therefore the aim of our research was to assess the potential for differences in self-reported use of ‘herbals’ using two different questioning approaches; single closed question and a list of specific ‘herbals’ administered consecutively.

## Methods

Data assessing the use of CAM collected from women (332) attending for their mid-trimester (18–21 weeks) scan and women with a live birth admitted to the postnatal unit (557) at the Royal Aberdeen Maternity Hospital, North-East Scotland were combined. Data collection was completed in 2012 and study methods have been reported in detail elsewhere [26, 27]; brief study details are given for completeness.

Questionnaires were based on the findings of our systematic review assessing the quality of study methodologies used to derive data reporting CAM use during pregnancy ([17], S1 Questionnaire]. The questionnaire was written in English only and tested for face and content validity by a panel of researchers, healthcare professionals, pregnant and postpartum women prior to piloting. Amongst other items, the questionnaire utilised two different approaches to collect data on ‘herbals’ used.

The first of these was one closed question, which asked the participant to tick all of a possible 23 different Complementary and Alternative Therapies which they had used (S1 Table of Complementary and Alternative Therapies). In the antenatal and postnatal studies participants were asked to: “Please tell us if you have used any of the following Complementary and Alternative Therapies during the last three months?” The question was followed by the request to “Please tick all Complementary and Alternative Therapies that you have used. If you haven’t heard of some of the names before, don’t worry. For each of the Complementary and Alternative Therapies you have used, please tell us why you used it and how you heard about it (doctor, pharmacists, midwife, family friend, internet, magazine)”. At a later stage in the questionnaire, participants were presented with an extensive list (sourced from the Medicines and Healthcare products Regulatory Agency (MHRA, UK)) of 40 ‘herbals’ [28] (S2 Table of Herbal and Natural Products) and asked in the antenatal and postnatal studies to “Please tell us if you have used any of the following Herbal and Natural Products during the last three months?; The question was then followed by the request to “Please tick all Herbal and Natural Products that you have used. If you haven’t heard of some of the names before, don’t worry. For each of the Herbal and Natural Products you have used, please tell us why you used it and how you heard about it (doctor, pharmacists, midwife, family friend, internet, magazine).

Data were analysed using descriptive statistics and a two tailed Pearson’s Chi square test to determine associations between the two different question approaches and the proportion answering yes to ‘herbals’ use. A P value <0.05 was considered statistically significant.

### Ethics Statement

This research was approved by National Health Service North of Scotland Research Ethics Committee and National Health Service Grampian Research and Development Committee on June 27, 2011 (REC 11/ AL/0094). As the Ethics Committee required the survey questionnaires to be fully anonymous and returned directly by respondents in post-paid envelopes with no record of identifiable data, oral rather than written consent to participate was deemed appropriate.

## Results

Eight hundred and eighty-nine respondents completed the questionnaire giving an overall response rate was 71%. Respondent demographics are reported in Table 2. Of 889 respondents, only 4.3% (38) reported use of ‘herbals’ via the closed question. However, using the detailed list 39% (350) of respondents reported the use of one or more specific ‘herbals’. Therefore 312 (35%) respondents who reported ‘no’ to ‘herbals’ use via the closed question actually reported “yes” to the use of herbs, herbal medicines or herbal products via the detailed list. This difference is statistically significant, p<0.0001.

**Table 2:** Study population demographics (n = 889).

Age (years)	Percentage% (n)
15–24	15 (132)
25–34	63 (564)
≥35	22 (193)
**Living circumstances**	
With spouse, partner	85 (759)
Other	15 (130)
**Education**	
University	51 (448)
College	29 (253)
Secondary school	21 (186)
**Ethnic origin**	
White British	80 (708)
Other	20 (173)
**First pregnancy**	53 (471)
**Mothers’ Existing Medical conditions**	26.0 (231)
Asthma	11 (95)
Hypertension	4.3 (38)
Depression	2.6 (23)
Diabetes	2.8 (25)
Epilepsy	0.7 (6)
Others	8.9 (79)
**Concurrent prescribed medication**	46 (406)

**Fig 2:**
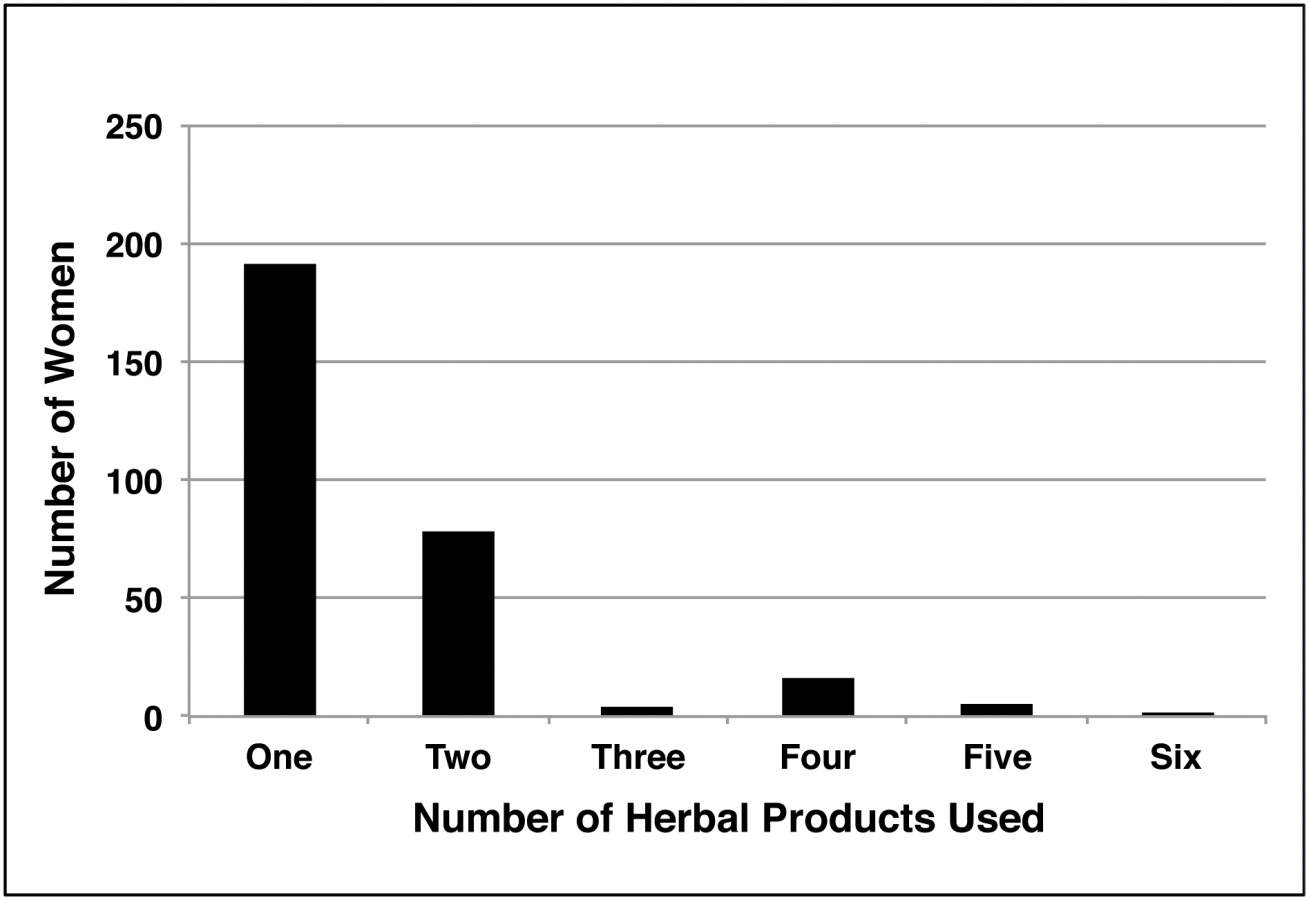
Bar chart of the actual number of different herbs, herbal medicines and herbal products taken or used by those responding ‘no’ to the closed question “have you used herbs, herbal medicines or herbal products in the last three months” (n = 312).

The 312 respondents who initially reported “no” actually consumed or used a total of 20 different ‘herbals’ (median 1, interquartile range 1–2, range 1–6) (See Fig 2).

The most frequently reported ‘herbals’ used were: raspberry tea or capsules 61% (126); ginger 29% (89); cranberry 22% (70); chamomile 16% (49); peppermint 12% (36); eucalyptus 8.3% (26); aloe 6.7% (21); grapefruit 6.4% (20); senna 5.4% (17); echinacea 4.5% (14); garlic beyond cooking 3.5% (11); ginseng 1.3% (4); 0.6% (2) each for aconite, nettle root, dong quai; 0.3% (1) each for barberry, bee pollen, blue cohosh, ginkgo biloba, and kava.

Binary logistic regression did not identify and significant differences, in terms of demographics, between the two study patient cohorts.

## Discussion

Our key finding is that using the closed question approach, as recommended in the I-CAM-Q [24, 25], grossly underestimated the true use of ‘herbals’ by the public. However employing a detailed list of ‘herbals’ generated a significant tenfold increase in the number of individuals reporting actual use.

To our knowledge this is the first study to compare two different approaches administered consecutively to the same cohort to determine the self-reported use of ‘herbals’.

In light of these findings there are key implications for the interpretation and generalizability for much of the research published in this area. It should not be surprising that data reported for the use of CAM modalities and ‘herbals’ during pregnancy are highly variable given that less than half of published prevalence studies described detailed checklists [17]. Of note, the I-CAM-Q asks respondents to name up to three herbs/herbal medicines taken or used in the past 12 months, therefore assuming that respondents can interpret the terms herbs and herbal medicines, and are able to categorise accordingly. Our data provide robust evidence that such an approach is likely to yield a gross and highly significant underestimate of true prevalence, threatening the internal validity of the data.

Population surveys such as the I-CAM-Q are routinely used to gain a picture of public practice or belief; however it is clear from the results of this study that two simple questions, which might at first glance be expected to give similar results, gave rise to significantly different responses. Although writing survey questions may initially appear simple the current literature would suggest otherwise [29–31]. The question setter must not only have a clear idea of the question intent and the likely responses, but also ensure that questions are written using language which the respondent can understand and process as intended [29–31]. Failure to follow the basic principles for questionnaire design and item writing described in the literature will inevitably give rise to misleading or erroneous survey results.

To ensure accurate and robust reporting of ‘herbals’ use during pregnancy the use of a detailed list is clearly necessary, however this may be problematic given that the number of available ‘herbals’ is increasing [32]. The development and application of specific checklists will require regular review and updating to acknowledge regional and temporal variability [32, 33] in ‘herbals’ use.

Although many ‘herbals’ have been used for centuries, underestimating the level of use during pregnancy is of clinical importance given that several of these agents have been directly associated with or have the potential to cause both maternal and fetal harm [5–16, 34–39].

### Strengths and Limitations

A key strength of this study is the consecutive administration of two different approaches to identify ‘herbals’ use in the same population.

A possible limitation however is the use of a study population in the North of Scotland, which may limit the generalizability of the findings. However our study population was relatively diverse in terms of ethnicity, parity and health status hence there is no reason to suspect that the outcome would be different in other populations. It is also possible that poor health literacy may have contributed to the significant differences we observed, however both approaches were administered concurrently to the study cohort and our population was relatively well educated, with over three quarters reporting a college or university education. Therefore it is unlikely that poor health literacy was responsible for the differences we observed between the approaches used.

## Conclusion

This study has demonstrated the need to ensure that detailed checklists of ‘herbals’ are used in all prevalence studies.

Our findings may also have relevance for the practicing clinician who should adopt detailed checklists when asking about a patient’s use of ‘herbals’.

## Supporting Information

S1 Questionnaire Exemplar Antenatal CAM in Pregnancy Questionnaire. (DOC)

S1 Table Table of Complementary and Alternative Therapies. Complementary and Alternative Therapies listed for the Question “Please tell us if you have used any of the following Complementary and Alternative Therapies?”
(DOCX)

S2 Table Table of Herbal Medicines and Products. Herbal Medicines and Products listed for the Question “Please tell us if you have used any of the following Herbal and Natural Products?
(DOCX)
