# Supplementary material for: 'Asking the Right Question'. A Comparison of Two Approaches to Gathering Data on 'Herbals' Use in Survey Based Studies
Source: PLoS One. 2016 Feb 25;11(2):e0150140. doi: 10.1371/journal.pone.0150140 (PMC4767213; doi:10.1371/journal.pone.0150140)
Supplement: S1 Questionnaire — (DOC) [file pone.0150140.s001.doc]

**Pregnancy and Complementary and Alternative Medicine Use**

Complementary and Alternative Medicine refers to the diagnosis, treatment and prevention of illness by various practitioners using therapies such as:

**herbal medicines**

**homopathic** **medicines**

**acupuncture**

**aromatherapy**

**chiropracty**

**vitamins and minerals**

**certain food products such as actimel, danone, benecol**

You are attending the clinic for a scan because you are expecting a baby. We are interested to find out whether you have used any Complementary and Alternative Medicine before or during your pregnancy. Thank you for taking the time to answer these questions. Please place the completed questionnaire in the reply-paid envelope supplied and either return to the clinic staff or send in the post.

**Section 1: This section contains some questions about you. Mark your answer with a (✓) in all the appropriate box or write in the space provided.**

1. How old are you?

2. What is your post-code (first half only)?    

3. Do you live with  Spouse or Partner  Other Family  Alone  Others ____________

4. Your education  Secondary school  College  University

5. How would you describe your ethnic origin?

 White British  Other white  Black British

 Other Black  Asian British  Other Asian

Chinese British  Other Chinese  Others ________________

**Section 2: This section is about your health. Mark your answer with a (✓) in all the appropriate boxes or write in the spaces provided. If you are not able to answer some questions, don’t worry.**

1. Is this your first pregnancy?

 Yes  No

2. How many weeks pregnant are you?

3. Do you have any of the following medical conditions?

 Diabetes  High blood pressure  Asthma Epilepsy  Depression

Other a) _______________ b) ______________ c) ____________

4. Have you taken any prescribed medicines during this pregnancy?

 Yes  No

If yes, please write the names of all the medicines you have been prescribed, even if only for a short time, in the space

below. Don’t worry if you can’t remember them all

1. _____________________________ f) _________________________
2. ____________________________ g) _________________________
3. _____________________________ h) __________________________
4. ______________________________ i) ___________________________
5. _____________________________ j) __________________________

**Section 3: This section has questions about your use of Complementary and Alternative Medicine.**

1. Please tell us if you have used any of the following Complementary and Alternative Medicine during **this** pregnancy?

**If you haven’t heard of some of the names before, don’t worry.**

For each of the Complementary and Alternative Medicine you have used, please tell us why you used it and how you heard about it (doctor, pharmacists, midwife, family friend, internet, magazine).

**(We have provided an example)**

| **Complementary and Alternative Medicine** | **Used during this pregnancy** | | **Why you used it** | **How you heard about it** |
| --- | --- | --- | --- | --- |
|  | ***Yes(✓)*** | ***No(✓)*** |  |  |
| ***Example: Massage*** | ***√*** |  | ***Back pain*** | ***Midwife*** |
| **Herbal Medicine** |  |  |  |  |
| **Homoeopathy** |  |  |  |  |
| **Chinese Medicine** |  |  |  |  |
| **Vitamins and Minerals** |  |  |  |  |
| **Aromatherapy** |  |  |  |  |
| **Massage** |  |  |  |  |
| **Nutraceutical (Lactobacillus drinks example : Danone actimel yoghurt drink)** |  |  |  |  |
| **Acupuncture** |  |  |  |  |
| **Acupressure** |  |  |  |  |
| **Chiropractic** |  |  |  |  |
| **Reiki** |  |  |  |  |
| **Ayurveda** |  |  |  |  |
| **Hypnosis** |  |  |  |  |
| **Shiatsu** |  |  |  |  |
| **Reflexology** |  |  |  |  |
| **Osteopathy** |  |  |  |  |
| **Spiritual Healing/Prayer** |  |  |  |  |
| **Alexander Technique** |  |  |  |  |
| **Cranial Osteopathy** |  |  |  |  |
| **Meditation** |  |  |  |  |
| **Yoga** |  |  |  |  |
| **Applied Kinesiology** |  |  |  |  |
| **Autogenic Training** |  |  |  |  |
| **Others:**  **a) ________________**  **b) ________________**  **c) ________________** |  |  |  |  |

2. Please tell us if you have used any of the following Herbal and Natural Products during **this** pregnancy?

**If you haven’t heard of some of the names before, don’t worry.**

For each of the Herbal and Natural Products you have used, please tell us why you used it and how you heard about it (doctor, pharmacists, midwife, family friend, internet, magazine).

**(We have provided an example)**

| **Herbal and natural Products** | **Used during this pregnancy** | | **Why you used it** | **How you heard about it** |
| --- | --- | --- | --- | --- |
|  | ***Yes(✓)*** | ***No(✓)*** |  |  |
| ***Example: Ginger*** | ***✓*** |  | ***Feeling Sick*** | ***Midwife*** |
| **Aloe** |  |  |  |  |
| **Echinacea** |  |  |  |  |
| **Ginseng** |  |  |  |  |
| **Barberry** |  |  |  |  |
| **St. John’s Wort** |  |  |  |  |
| **Black Cohosh** |  |  |  |  |
| **Kelp** |  |  |  |  |
| **Cat's Claw** |  |  |  |  |
| **Ephedra** |  |  |  |  |
| **Tansy** |  |  |  |  |
| **Rue** |  |  |  |  |
| **Yarrow** |  |  |  |  |
| **Nettle root** |  |  |  |  |
| **Baldo** |  |  |  |  |
| **Goldenseal** |  |  |  |  |
| **Comfrey** |  |  |  |  |
| **Blue Cohosh** |  |  |  |  |
| **Clove Oil** |  |  |  |  |
| **Dong Quai** |  |  |  |  |
| **Wormwood** |  |  |  |  |
| **Senna** |  |  |  |  |
| **Ginkgo Biloba** |  |  |  |  |
| Ginseng |  |  |  |  |
| **Tea-tree oil** |  |  |  |  |
| **Eucalyptus** |  |  |  |  |
| **Glucosamine** |  |  |  |  |
| **Aconite** |  |  |  |  |
| **Bee Pollen** |  |  |  |  |
| **Evening Primrose** |  |  |  |  |
| **Milk Thistle** |  |  |  |  |
| **Grapefruit** |  |  |  |  |
| **Chamomile** |  |  |  |  |
| **Garlic *(beyond cooking)*** |  |  |  |  |
| **Ginger *(beyond cooking)*** |  |  |  |  |
| **Fish Oil (omega 3)** |  |  |  |  |
| **Coenzyme Q10** |  |  |  |  |
| **Cranberry** |  |  |  |  |
| **Kava** |  |  |  |  |
| **Cod-liver Oil** |  |  |  |  |
| **Squill** |  |  |  |  |
| **Others**   1. **____________** 2. **____________** 3. **____________** |  |  |  |  |

3. Please tell us if you have used any of the following Essential oils for Aromatherapy during **this** pregnancy?

**If you haven’t heard of some of the names before, don’t worry.**

For each of the Essential oils you have used, please tell us why you used it and how you heard about it

(doctor, pharmacists, midwife, family friend, internet, magazine).

**(We have provided an example)**

| **Essential oils for Aromatherapy** | **Used during this pregnancy** | | **Why you used it** | **How you heard about it** |
| --- | --- | --- | --- | --- |
|  | ***Yes(✓)*** | ***No(✓)*** |  |  |
| ***Example: Peppermint*** | ***✓*** |  | ***asthma*** | ***Midwife*** |
| **Basil** |  |  |  |  |
| **Cedarwood** |  |  |  |  |
| **Clary** |  |  |  |  |
| **Hyssop** |  |  |  |  |
| **Coriander** |  |  |  |  |
| **Jasmine** |  |  |  |  |
| **Juniper** |  |  |  |  |
| **Marjoram** |  |  |  |  |
| **Rosemary** |  |  |  |  |
| **Peppermint** |  |  |  |  |
| **Myrrh** |  |  |  |  |
| **Thyme** |  |  |  |  |
| **Rockrose** |  |  |  |  |
| **Sage** |  |  |  |  |
| **Lavender** |  |  |  |  |
| **Others**   1. **__________** 2. **__________** 3. **__________** |  |  |  |  |

4: Please tell us if you have used any Vitamin and Mineral supplements during **this** pregnancy? For each of the Vitamin and Mineral supplements you have used, please tell us why you used it and how you heard about it (doctor, pharmacists, midwife, family friend, internet, magazine).

**(We have provided an example)**

| **Vitamin and mineral supplements (Trade name)** | **Used during this pregnancy** | | **Why you used it** | **How you heard about it** |
| --- | --- | --- | --- | --- |
|  | ***Yes(✓)*** | ***No(✓)*** |  |  |
| ***Example: Pregaday*** | ***✓*** |  | ***vitamin supplement*** | ***Midwife*** |
|  |  |  |  |  |
|  |  |  |  |  |
|  |  |  |  |  |
|  |  |  |  |  |
|  |  |  |  |  |
|  |  |  |  |  |

5: Were you using any Complementary and Alternative Medicine before you became pregnant?

 Yes  No

1. _______________________ c) ______________________
2. ________________________ d) ______________________

If yes, what did you use?

6: Did you start to use any Complementary and Alternative Medicine on becoming pregnant?

 Yes  No

If yes, tell us why?

7: Did you stop using any Complementary and Alternative Medicine on becoming pregnant?

 Yes  No

If yes, tell us why?

8: Does anyone you know use Complementary and Alternative Medicine? (✓ more than one if appropriate)

 Spouse or Partner  Family member  Friend  None  Other (please tell us) _________

**Section 4: This section has statements relating to Complementary and Alternative Medicine use**

**For each statement below, put a (✓) in the box that best describes how much you agree or disagree.**

**There are no right or wrong answers.**

| **Statements** | **Strongly**  **Agree** | **Agree** | **Uncertain** | **Disagree** | **Strongly Disagree** |
| --- | --- | --- | --- | --- | --- |
| During pregnancy Complementary and Alternative Medicine are safer than conventional medicines prescribed by my doctor |  |  |  |  |  |
| Complementary and Alternative Medicine are more effective than conventional medicines prescribed by my doctors during pregnancy |  |  |  |  |  |
| Complementary and Alternative Medicine can interfere with conventional medicines prescribed by my doctors |  |  |  |  |  |
| Complementary and Alternative Therapies can cause side effects |  |  |  |  |  |
| Health care professionals should be informed by patients about the use of any Complementary and Alternative Medicine during pregnancy |  |  |  |  |  |
| Complementary and Alternative Medicine should be available through the NHS |  |  |  |  |  |

**Thank you for completing this questionnaire**

**Please return to the scan clinic receptionists in the envelop or send back to us in the reply paid envelope**

ABERDEEN RGU HEADED PAPER

Centre Number:

Study Number:

Participant Identification Number for this study:

Please complete both copies of this consent form if you would like to help us by:

- Completing a further questionnaire on Complementary and Alternative Therapies after the birth of your baby

**CONSENT FORM**

**Pregnancy and Complementary and Alternative Medicine Use**

**Please initial each box**

1. I have read and understand the information sheet dated ............ ⬜

(Version 1.0) for the above study.

2. I understand that taking part is voluntary and that I can withdraw ⬜

at any time, without giving any reason, without my legal rights being affected.

3. I agree that you can contact me about a further questionnaire at my address below. ⬜

________________________ ________________ ____________________

Name of Participant Date Signature

Address:

____________________________________________

____________________________________________

One copy to be kept by you, one to be returned with this questionnaire:

Name of lead researcher: Dr James McLay

If you have any questions please contact

Dr Ashalatha Shetty, Consultant Obstetrician

Aberdeen Maternity Hospital Foresterhill,

Aberdeen AB25 2ZL

Tel: +44 (0)1224 552606

Email: [a.shetty@abdn.ac.uk](mailto:a.shetty@abdn.ac.uk)

Bleep: 2352
