# Supplementary material for: 'Asking the Right Question'. A Comparison of Two Approaches to Gathering Data on 'Herbals' Use in Survey Based Studies
Source: PLoS One. 2016 Feb 25;11(2):e0150140. doi: 10.1371/journal.pone.0150140 (PMC4767213; doi:10.1371/journal.pone.0150140)
Supplement: S1 Table — Complementary and Alternative Therapies listed for the Question “Please tell us if you have used any of the following Complementary and Alternative Therapies?” (DOCX) [file pone.0150140.s002.docx]

| **Complementary and Alternative Therapy** | **Used during the last half of your pregnancy** | | **Why you used it** | **How you heard about it** |
| --- | --- | --- | --- | --- |
|  | ***Yes(✓)*** | ***No(✓)*** |  |  |
| ***Example: Massage*** | ***√*** |  | ***Back pain*** | ***Midwife*** |
| **Herbal Medicine** |  |  |  |  |
| **Homeopathy** |  |  |  |  |
| **Chinese Medicine** |  |  |  |  |
| **Vitamins and Minerals** |  |  |  |  |
| **Aromatherapy** |  |  |  |  |
| **Massage** |  |  |  |  |
| **Nutraceutical (Lactobacillus drinks example : Danone actimel yoghurt drink)** |  |  |  |  |
| **Acupuncture** |  |  |  |  |
| **Acupressure** |  |  |  |  |
| **Chiropractic** |  |  |  |  |
| **Reiki** |  |  |  |  |
| **Ayurveda** |  |  |  |  |
| **Hypnosis** |  |  |  |  |
| **Shiatsu** |  |  |  |  |
| **Reflexology** |  |  |  |  |
| **Osteopathy** |  |  |  |  |
| **Spiritual Healing/Prayer** |  |  |  |  |
| **Alexander Technique** |  |  |  |  |
| **Cranial Osteopathy** |  |  |  |  |
| **Meditation** |  |  |  |  |
| **Yoga** |  |  |  |  |
| **Applied Kinesiology** |  |  |  |  |
| **Autogenic Training** |  |  |  |  |
| **Others:**  **a)**  **b)**  **c)** |  |  |  |  |
