# Supplementary material for: 'Asking the Right Question'. A Comparison of Two Approaches to Gathering Data on 'Herbals' Use in Survey Based Studies
Source: PLoS One. 2016 Feb 25;11(2):e0150140. doi: 10.1371/journal.pone.0150140 (PMC4767213; doi:10.1371/journal.pone.0150140)
Supplement: S2 Table — Herbal Medicines and Products listed for the Question “Please tell us if you have used any of the following Herbal and Natural Products? (DOCX) [file pone.0150140.s003.docx]

| **Herbal and natural Products** | **Used during the last half of your pregnancy** | | **Why you used it** | **How you heard about it** |
| --- | --- | --- | --- | --- |
|  | ***Yes(✓)*** | ***No(✓)*** |  |  |
| ***Example: Ginger*** | ***✓*** |  | ***Feeling Sick*** | ***Midwife*** |
| **Aloe** |  |  |  |  |
| **Echinacea** |  |  |  |  |
| **Ginseng** |  |  |  |  |
| **Barberry** |  |  |  |  |
| **St. John’s Wort** |  |  |  |  |
| **Black Cohosh** |  |  |  |  |
| **Kelp** |  |  |  |  |
| **Cat's Claw** |  |  |  |  |
| **Ephedra** |  |  |  |  |
| **Tansy** |  |  |  |  |
| **Rue** |  |  |  |  |
| **Yarrow** |  |  |  |  |
| **Nettle root** |  |  |  |  |
| **Baldo** |  |  |  |  |
| **Goldenseal** |  |  |  |  |
| **Comfrey** |  |  |  |  |
| **Blue Cohosh** |  |  |  |  |
| **Clove Oil** |  |  |  |  |
| **Dong Quai** |  |  |  |  |
| **Wormwood** |  |  |  |  |
| **Senna** |  |  |  |  |
| **Ginkgo Biloba** |  |  |  |  |
| **Ginseng** |  |  |  |  |
| **Tea-tree oil** |  |  |  |  |
| **Eucalyptus** |  |  |  |  |
| **Glucosamine** |  |  |  |  |
| **Aconite** |  |  |  |  |
| **Bee Pollen** |  |  |  |  |
| **Evening Primrose** |  |  |  |  |
| **Milk Thistle** |  |  |  |  |
| **Grapefruit** |  |  |  |  |
| **Chamomile** |  |  |  |  |
| **Garlic *(beyond cooking)*** |  |  |  |  |
| **Ginger *(beyond cooking)*** |  |  |  |  |
| **Fish Oil (omega 3)** |  |  |  |  |
| **Coenzyme Q10** |  |  |  |  |
| **Cranberry** |  |  |  |  |
| **Kava** |  |  |  |  |
| **Cod-liver Oil** |  |  |  |  |
| **Squill** |  |  |  |  |
| **Others**   1. **____________** 2. **____________** 3. **____________** |  |  |  |  |
